# Gender‐Affirming Hormone Treatment Decreases Bone Turnover in Transwomen and Older Transmen

**DOI:** 10.1002/jbmr.3762

**Published:** 2019-08-19

**Authors:** Mariska C Vlot, Chantal M Wiepjes, Renate T de Jongh, Guy T’Sjoen, Annemieke C Heijboer, Martin den Heijer

**Affiliations:** ^1^ Department of Internal Medicine and Center of Expertise on Gender Dysphoria Amsterdam UMC, Vrije Universiteit Amsterdam, A msterdam The Netherlands; ^2^ Department of Clinical Chemistry Endocrine laboratory, Amsterdam UMC, Vrije Universiteit Amsterdam Amsterdam The Netherlands; ^3^ Department of Endocrinology Center for Sexology and Gender, Ghent University Hospital Ghent Belgium

**Keywords:** TRANSGENDER, GENDER‐AFFIRMING HORMONE TREATMENT, BONE TURNOVER MARKER, SCLEROSTIN, P1NP

## Abstract

Sex steroids play a key role in bone turnover and preserving BMD; hence, gender‐affirming hormone treatment (HT) in transgender people affects bone metabolism. Most studies have looked into the effect of HT on changes in BMD; however, they do not provide insights into changes in bone metabolism caused by HT. This study investigated changes in bone turnover markers (BTMs) and sclerostin, as well as their correlations with change in BMD in transwomen and transmen during the first year of HT. Transwomen received estradiol and antiandrogens; transmen received testosterone. Sclerostin; P1NP; alkaline phosphatase (ALP); CTx; and BMD of the total hip, the femoral neck, and the lumbar spine were evaluated at baseline and after 1 year of HT. There were 121 transwomen (median age 30 years, interquartile range [IQR] 24 to 41 years) and 132 transmen (median age 24 years, IQR 21 to 33 years) included in the study. In transwomen, ALP decreased in 19% (95% CI, –21 to–16), CTx in 11% (95% CI, –18 to–4), and sclerostin in 8% (95%CI, –13 to–4) of study participants after 1 year of HT. In contrast, in transmen P1NP, ALP, and sclerostin increased in 33% (95% CI, 24 to 42), 16% (95% CI, 12 to 20), and 15% (95% CI, 10 to 20) of study participants, respectively, after 1 year of HT. No age differences were seen in transwomen, whereas in transmen aged ≥50 years a decrease in all BTMs was found in contrast with the other age groups. These transmen had low estrogen concentration at the start of HT based on their postmenopausal state before the start of HT; their estradiol concentrations increased during testosterone treatment. Changes in BTMs and BMD were weakly correlated (correlation coefficient all <0.30). To conclude, 1 year of HT resulted in decreased bone turnover in transwomen and older transmen, whereas it increased in younger transmen. The decrease in bone resorption in older transmen shows the importance of estrogen as a key regulator of bone turnover. © 2019 The Authors. *Journal of Bone and Mineral Research* published by Wiley Periodicals, Inc.

## Introduction

Sex steroids are considered to be pivotal regulators of bone metabolism. Estrogen inhibits the osteoclast function and thereby lowers bone resorption, resulting in a positive effect on BMD in both women and men.^1^, ^2^, ^3^, ^4^, ^5^ Furthermore, it is well‐known that BMD decreases in postmenopausal women based on decreasing estrogen concentrations and consequently increased bone resorption by osteoclasts.^6^, ^7^ In men, estrogen is aromatized from testosterone and is also considered to be the key sex steroid affecting bone homeostasis.^8^, ^9^, ^10^ Research has shown that bone metabolism and therefore BMD are affected by gender‐affirming hormone treatment (HT) in people diagnosed with gender dysphoria^5^, ^11^, ^12^, ^13^, ^14^, ^15^, ^16^, ^17^, ^18^: HT is used to bring about desired body changes in transgender people. HT in transmen (female‐to‐male transgender people) consists of testosterone treatment; transwomen (male‐to‐female transgender people) receive a combination of antiandrogens and estrogens.

An increase in BMD after 1 to 10 years of treatment with HT in transgender people has been described.^5^, ^19^ BMD is usually evaluated by a DXA scan; however, these scans estimate the amount of mineralized bone only and therefore represent late changes in bone metabolism. In contrast, bone turnover markers (BTMs) represent the actual activity of the osteoblasts and osteoclasts. Consequently, measurements of BTMs display the balance between bone formation and bone resorption directly. Up until now, scarce data have been available regarding the effect of HT on bone turnover specifically in transgender people,^20^, ^21^, ^22^, ^23^, ^24^ whereas no data on sclerostin are available yet. Clinically, increased bone turnover and lower BMD are risk factors for the deterioration of bone quality, resulting in possible osteopenia, osteoporosis, and even an increased risk of fractures with its associated comorbidities and financial costs. As the transgender population receiving HT increases worldwide,^25^, ^26^ more transgender people are thus at risk for lower bone quality and its associated problems. Therefore, the aim of this study was to investigate the change in BTMs and to evaluate the correlations with changes in BMD in adult transgender people during their first year of HT. We also studied possible age‐related effects on BTMs and BMD during HT by studying transgender people in various age groups. We hypothesized that we would find a decrease in bone turnover predominantly caused by estrogen, as this sex steroid is known to inhibit osteoclast function and therefore exerts an anabolic effect on bone.

This study also focused on the bone formation markers P1NP and total alkaline phosphatase (ALP) and the bone resorption marker CTx. In addition, we studied the glycoprotein sclerostin, which is known^1^: to mediate an antianabolic effect on bone by promoting the apoptosis of osteoblasts^2^; to stimulate RANKL production by osteocytes, resulting in increased osteoclastogenesis; and 3 to inhibit the activation of the Wnt/β‐catenin pathway— all resulting in negative effects on BMD.^27^, ^28^, ^29^, ^30^, ^31^, ^32^ Sclerostin is mainly produced by osteocytes and can be used as a marker of bone metabolism as well.

## Methods

### Subjects and study protocol

This study is part of the European Network for Investigation of Gender Incongruence (ENIGI) study, which is a prospective multicenter observational study in Ghent (Belgium), Oslo (Norway), Florence (Italy), and Amsterdam (The Netherlands).^33^, ^34^ The current study protocol was approved by the Ethical Committee of the Amsterdam University Medical Center, Vrije Universiteit Amsterdam (Amsterdam, The Netherlands). Data were retrieved only after informed consent. Adults diagnosed with gender dysphoria based on the diagnostic criteria of the *Diagnostic and Statistical Manual of Mental Disorders*, 4th edition (*DSM IV*)^35^ or 5^th^ edition (*DSM5)* 36 were recruited at the Center of Expertise on Gender Dysphoria of the Amsterdam University Medical Center, between June 2012 and April 2016. All transgender people included in this study were treated according to the Standards of Care Guidelines of the World Professional Association for Transgender Health (WPATH).^37^ Transwomen were treated with antiandrogen treatment consisting of cyproterone acetate (50 to 100 mg daily, oral) accompanied by estrogen treatment consisting of either estradiol valerate (2 to 4 mg daily, oral) or estradiol patches (50 to 100 µg/24 hours twice a week, transdermal application). Transmen were treated with either testosterone gel (50 mg daily, dermal application), testosterone esters (250 mg every 2 to 3 weeks, i.m.), or testosterone undecanoate (1000 mg every 12 weeks, i.m.). Some transmen used lynestrenol for a short period if menses persisted while using testosterone.

People were not eligible to participate in the study if they had^1^: insufficient knowledge of their native language^2^; were psychologically vulnerable^3^; used HT earlier in life; or 4 used other drug therapies that were not part of the standardized treatment protocol (eg, spironolactone or gonadotropin‐releasing hormone agonists). For the current analyses, people were excluded if they did not complete 1 year of HT, had no DXA scan at baseline and/or after 1 year of HT, or had no blood drawn at baseline and after 1 year of HT. In addition, only people from the Amsterdam University Medical Center were included to exclude possible changes in BTM concentrations caused by the use of different BTM assays in other medical centers.

### Measurements

#### General

Participants visited the outpatient clinic every 3 months to evaluate their health and treatment effects. Body weight (kilograms) and height (centimeters) were measured without wearing shoes at baseline and follow‐up. Blood samples were collected between 9:00 a.m. and 12:00 p.m. at baseline, after 3 months, and after 1 year of HT. Participants were instructed to draw blood in a fasting state.

#### Bone turnover markers

##### P1NP

The bone formation marker P1NP resembles osteoblast activity^38^ and was measured using an immunoassay (Cobas; Roche Diagnostics, Mannheim, Germany), with an interassay coefficient of variation (CV) of <8% and a lower limit of quantification (LOQ) of 5 µg/L.

##### Alkaline phosphatase

The bone formation marker ALP, also representing osteoblast activity,^38^ was measured using an immunoassay (Cobas; Roche Diagnostics), with an interassay CV of 2.5% and a LOQ of 5 U/L.

##### CTx

The bone resorption marker CTx displays osteoclast activity.^38^ CTx was measured using an immunoassay (Cobas; Roche Diagnostics), with an interassay CV of <6.5% and a LOQ of 10 ng/L.

##### Sclerostin

The osteocyte‐derived glycoprotein sclerostin^38^ was measured using an immunoassay (LiasonXL; Diasorin, Saluggia, Italy), with an interassay CV of 7.5% and a LOQ of 2.2 pmol/L.

#### Other measurements

##### 25OHD

Liquid chromatography–tandem mass spectrometry (LC‐MS/MS) was used to measure 25OHD with a CV of 8% and a LOQ of 4.0 nmol/L until 2015.^39^ From then on, another LC‐MS/MS method was used.^40^ Both methods resulted in comparable concentrations.

##### Testosterone

A radioimmunofrequent assay (RIA; Coat‐A‐Count; Siemens Medical Solutions USA, Malvern, PA, USA; an interassay CV of 7% to 20%, a LOQ of 1 nmol/L) was used to measure testosterone until January 2013. From then on, a competitive immunoassay was used (Architect; Abbott Laboratories, Abbott Park, IL, USA; an interassay CV of 6% to 10%, a LOQ of 0.1 nmol/L). The RIA‐based concentrations were converted to concentrations of the competitive immunoassay using the formulas: Architect = 1.1 * RIA + 0.20 (for testosterone concentrations <8 nmol/L) and Architect = 1.34 * RIA – 1.65 (for testosterone concentrations >8 nmol/L) to evaluate and report comparable testosterone concentrations.

##### Estradiol

A competitive immunoassay (Delfia; PerkinElmer, Turku, Finland; interassay CV of 10% to 13%, LOQ of 20 pmol/L) was used to measure estradiol until July 2014. Subsequently, an LC‐MS/MS (Amsterdam University Medical Center; an interassay CV of <7%, a LOQ of 20 pmol/L) was used. The Delfia concentrations were converted to the LC‐MS/MS concentrations by using the formula LC‐MS/MS = 1.60 * Delfia – 29.

Creatinine, aspartate transaminase (AST), alanine transaminase (ALT), and gamma‐glutamyltransferase (γGT) concentrations were all measured using an immunoassay (Cobas; Roche Diagnostics).

#### DXA

DXA (Hologic Discovery A; Hologic Inc., Bedford, MA, USA) was used to measure BMD in g/cm^2^ of the total hip (TH) and femoral neck (FN) of the nondominant hip and the lumbar spine (LS), measuring the first four lumbar vertebrae (L1 to L4). The software was updated from version 13.3 to 13.5.3 in July 2015, which did not affect the results of the measurements. Baseline DXA was performed 3 months before to 1 month after the start of HT. The follow‐up DXA was performed between 10 and 14 months after the start of HT.

### Statistics

For statistical analyses, Stata/SE 15 (StataCorp, LP, College Station, TX, USA) was used. The median with corresponding interquartile range (IQR), percentages, or means with SD were used to describe baseline characteristics. The percentage change was calculated for all BTMs and BMDs to evaluate differences between baseline and 1‐year HT. As these changes were normally distributed, linear regression analyses were performed to evaluate mean changes in percentage with corresponding 95% CI. Next, these percentage difference variables were adjusted for changes in BMI, alcohol and tobacco use, 25OHD, creatinine, AST, ALT, and γGT concentrations. Participants were stratified for both age and sex steroid concentrations, with the following age groups: 18 to 30 years, 30 to 50 years, and ≥50 years. By using these separate age groups, age‐related differences in BMD caused by decreasing bone mass with increasing age after reaching peak bone mass is accounted for; it is expected that bone mass decreases throughout time as described.^5^ Linear regression was performed to evaluate possible differences between the separate age groups. Furthermore, participants were stratified into quartiles based on their mean estradiol and testosterone concentrations during HT, which were calculated by an average of the concentrations after 3 and 12 months of HT. This stratification was applied to detect possible differences between the effects of either low or high sex steroid concentrations. Furthermore, a power analysis was performed. The analysis was applied to the study population of 121 transwomen and 132 transmen to detect the mean differences of both BMD and separate BTMs with a power of 80% and alpha of 0.05. This resulted in the detection of a mean difference of LS BMD of 0.021 g/cm^2^ in transwomen and 0.022 g/cm ^2^ in transmen. Regarding BTMs, in transwomen a 10% change in CTx, 9% change in P1NP, 4% change in ALP, and 6% change in sclerostin could be detected. In transmen, a change of 10%, 13%, 6%, and 7% could be detected, respectively. Pearson correlations were calculated between the change in BTMs and BMD and are displayed with corresponding 95% CI (Table 3).

## Results

### General

**Figure 1:**
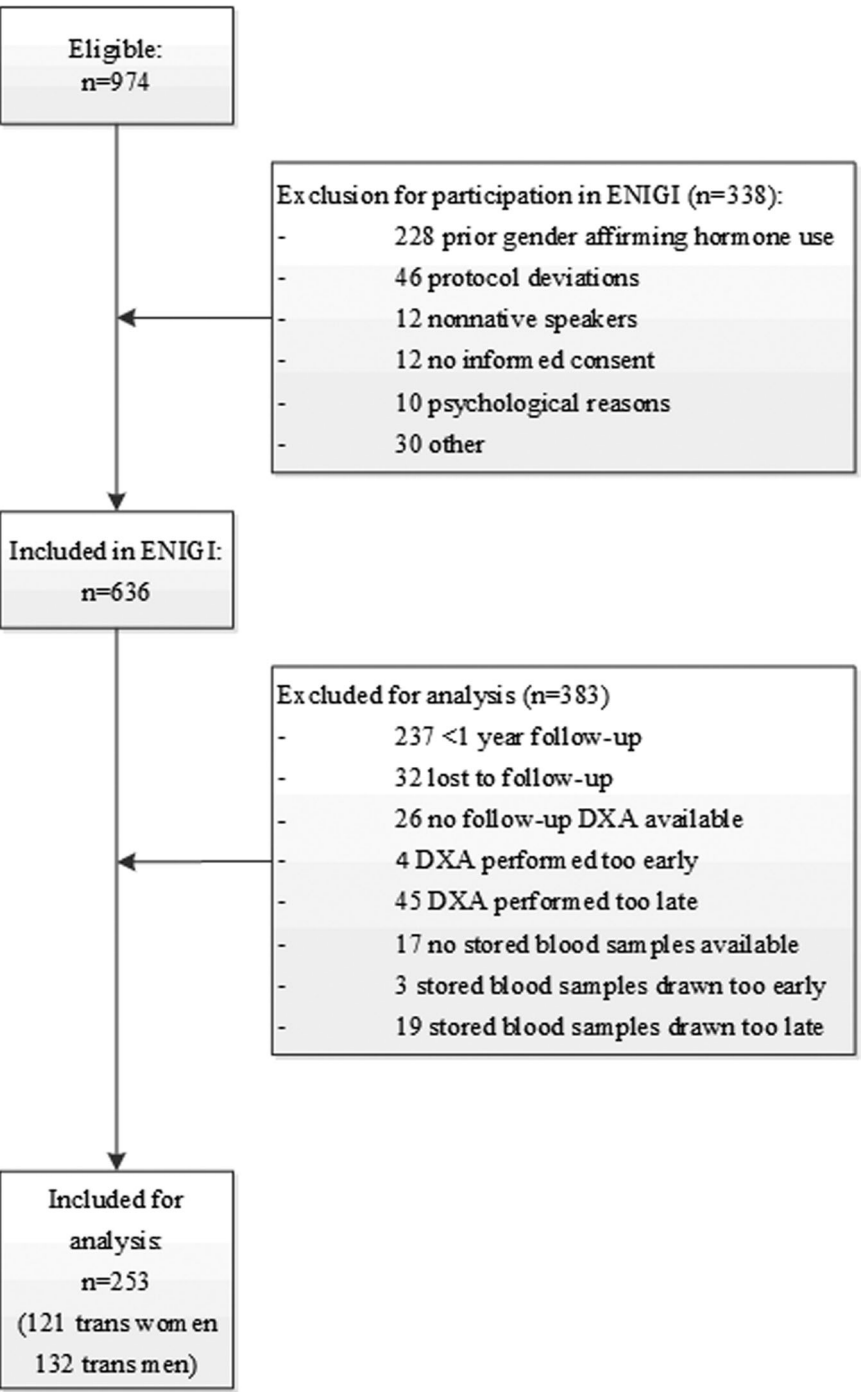
Flowchart of inclusion and exclusion of participants. ENIGI = European Network for Investigation of Gender Incongruence.

A total of 253 people were included in this study (Fig. 1), which consisted of 121 transwomen with a median age of 30 (IQR 24 to 41) years and 132 transmen with a median age of 24 (IQR 21 to 33) years. The baseline and follow‐up characteristics are displayed in Table 1. In transwomen, a median increase in estradiol of 129 pmol/L (IQR 56 to 232) implying a percentage change of estradiol of 128% (IQR 52 to 214) and a median decrease in testosterone of –18 nmol/L (IQR –22 to –14) with a percentage decrease of –96% (IQR –97 to –94) was seen during the first year of HT. In transmen, a median increase in estradiol of 46 pmol/L (IQR –304 to 135) with a percentage change of 26% (IQR –63 to 198), which was accompanied by a median increase in testosterone of 27 nmol/L (IQR 20 to 38) and a percentage increase of 2248% (IQR 1311 to 3338) was seen during the first year of HT.

**Table 1:** Participant Characteristics at Baseline and After 1 Year of HT

	Transwomen (*n* = 121)		Transmen (*n* = 132)	
	Baseline	1 year of HT	Baseline	1 year of HT
General				
Age, year (median, IQR)	30 (24 to 41)		24 (21 to 33)	
Ethnicity (% white)	96.7		91.7	
BMI, kg/m^2^ (median, IQR)	22.9 (20.8 to 26.1)	24.1 (21.9 to 26.3)	24.5 (21.4 to 29.0)	25.4 (22.5 to 29.2)
Tobacco use (% yes)	24.0	14.0	29.2	20.0
Cigarettes/day (median, IQR)	10 (5 to 10)	6 (4 to 20)	8 (4 to 15)	7 (3 to 15)
Alcohol use (% yes)	46.3	45.6	51.2	47.5
Units/week (median, IQR)	2 (1 to 5)	2 (2 to 4)	2 (1 to 4)	3 (2 to 5)
Biochemical results (median, IQR)				
Estradiol, pmol/L	105 (84 to 133)	204 (137 to 328)	187 (67 to 525)	181 (132 to 261)
Testosterone, nmol/L	19.0 (14.0 to 23.0)	0.7 (0.5 to 1.0)	1.3 (1.0 to 1.7)	29 (20 to 39)
LH, U/L	3.2 (2.3 to 4.3)	0.1 (0.1 to 0.1)	5.0 (2.7 to 6.9)	1.5 (0.2 to 3.6)
25OHD, nmol/L	39 (25 to 57)	60 (40 to 76)	54 (30 to 77)	57 (41 to 80)
Creatinine, µmol/L (mean ± SD)	77 ± 10	73 ± 10	66 ± 10	79 ± 12
AST, U/L	24 (20 to 28)	20 (17 to 23)	21 (19 to 25)	24 (20 to 28)
ALT, U/L	22 (16 to 30)	21 (15 to 27)	17 (13 to 24)	22 (17 to 29)
γGT, U/L	20 (15 to 28)	19 (15 to 26)	15 (12 to 23)	17 (12 to 26)

HT = gender‐affirming hormonal treatment; IQR = interquartile range; LH = luteinizing hormone; AST = aspartate transaminase; ALT = alanine transaminase; γGT = gamma‐glutamyltransferase.

In both groups, the BMI increased and tobacco use decreased during 1 year of HT (Table 1).

### Transwomen

**Figure 2:**
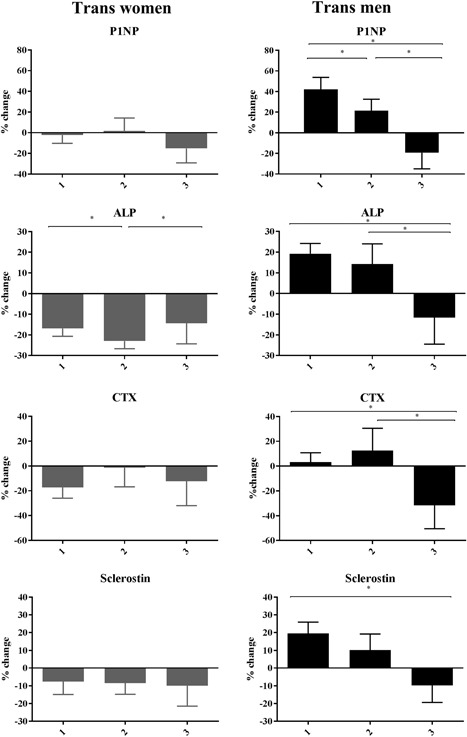
Percentage change in bone turnover markers in transwomen and transmen after 1 year of hormone therapy, stratified by age groups. Group 1 = 18 to 30 years [transwomen mean age 24 (2.9 SD), *n* = 61, transmen mean age 23 (3.0 SD), *n* = 91]. Group 2 = 30 to 50 years [transwomen mean age 39 (5.2 SD), *n* = 42, transmen mean age 39 (5.9 SD), *n* = 32]. Group 3 = group ≥50 years [transwomen mean age 56 (5.8 SD), *n* = 18, transmen mean age 54 (4.1 SD), *n* = 9]. ALP = alkaline phosphatase. *p ≤ 0.05.

**Figure 3:**
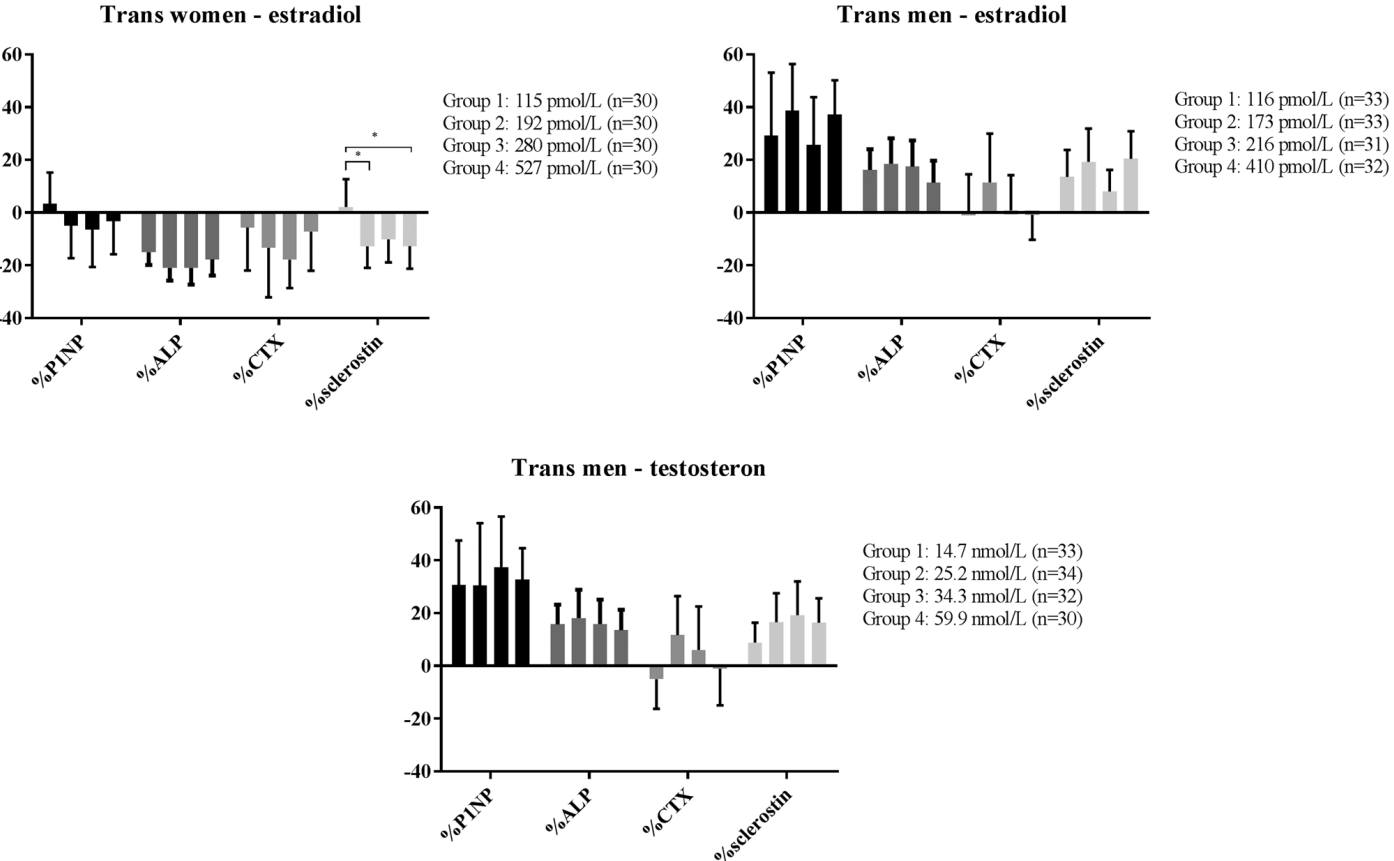
Percentage change in bone turnover markers by quartiles of average estradiol and testosterone concentrations measured at 3 and 12 months after baseline. Testosterone concentrations in transwomen were <2 nmol/L; therefore, this group was not further divided into subgroups. ALP = alkaline phosphatase. *p ≤ 0.05.

ALP, CTx, and sclerostin decreased by 19% (95% CI, –21 to –16), 11% (95% CI, –18 to –4), and 8% (95% CI, –13 to –4), respectively, in the unadjusted model after 1 year of HT (Table 2). Adjusting the percentage changes in all BTMs for changes in BMI, smoking habits, alcohol use, 25OHD, creatinine, AST, ALT, and γGT concentrations did not affect the results (Table 2). No difference between the different age groups in change in BTMs was found (Fig. 2). Sclerostin decreased in all, but the lowest estradiol quartile (Fig. 3).

**Table 2:** Baseline and 1‐Year Concentrations of Bone Turnover Markers and BMD With Corresponding Percentage Change (Mean and 95% CI), for Transwomen and Transmen Separately

	Baseline	1‐year HT	Percentage change %	Percentage change % adjusted^a^
Transwomen				
Bone turnover markers				
P1NP, µg/L (median, IQR)	50 (42 to 65)	48 (38 to 62)	–3 (–9 to 3)	–8 (–17 to 1)
18 to 30 years	61 (49 to 74)	52 (47 to 75)	–2 (–10 to 6)	n.a.
30 to 50 years	48 (38 to 52)	46 (35 to 54)	+2 (–10 to 14)	n.a.
≥50 years	40 (33 to 43)	29 (22 to 39)	–15 (–29 to –1)	n.a.
ALP, U/L (mean ± SD)	70 ± 17	57 ± 17	–19 (–21 to–16)	–21 (–25 to 18)
18 to 30 years	72 ± 19	60 ± 18	–17 (–21 to –13)	n.a.
30 to 50 years	69 ± 16	53 ± 13	–23 (–27 to –19)	n.a.
≥50 years	67 ± 13	58 ± 19	–14 (–24 to–4)	n.a.
CTx, ng/L (median, IQR)	428 (306 to 538)	329 (265 to 442)	–11 (–18 to–4)	–11 (–23 to 1)
18 to 30 years	507 (387 to 658)	351 (309 to 476)	–17 (–26 to –9)	n.a.
30 to 50 years	371 (275 to 500)	313 (265 to 452)	–1 (–17 to 14)	n.a.
≥50 years	287 (198 to 369)	224 (165 to 279)	–12 (–32 to 7)	n.a.
Sclerostin, pmol/L (median, IQR)	10.4 (8.6 to 14.9)	8.8 (7.3 to 13.5)	–8 (–13 to –4)	–9 (–16 to –2)
18 to 30 years	8.8 (7.7 to 11.0)	7.7 (6.6 to 9.4)	–8 (–15 to 0)	n.a.
30 to 50 years	11.4 (9.4 to 15.0)	11.0 (8.1 to 13.4)	–9 (–15 to –2)	n.a.
≥50 years	17.7 (16.0 to 21.9)	17.9 (14.1 to 18.5)	–10 (–22 to 2)	n.a.
DXA				
BMD TH g/cm^2^ (mean ± SD)	0.938 ± 0.137	0.947 ± 0.137	+1.0 (0.5 to 1.5)	+0.8 (0.1 to 1.6)
BMD FN g/cm^2^ (mean ± SD)	0.797 ± 0.127	0.812 ± 0.129	+1.9 (1.3 to 2.5)	+1.6 (0.7 to 2.5)
BMD LS g/cm^2^ (mean ± SD)	0.968 ± 0.139	1.004 ± 0.138	+3.8 (3.1 to 4.6)	+3.2 (2.0 to 4.4)
Transmen				
Bone turnover markers				
P1NP, µg/L (median, IQR)	56 (43 to 71)	71 (49 to 100)	+33 (24 to 42)	+29 (11 to 48)
18 to 30 years	60 (50 to 77)	85 (67 to 111)	+42 (30 to 54)	n.a.
30 to 50 years	40 (36 to 52)	53 (37 to 60)	+21 (10 to 33)	n.a.
≥50 years	46 (41 to 66)	41 (29 to 55)	–19 (–35 to –4)	n.a.
ALP, U/L (mean ± SD)	67 ± 19	76 ± 22	+16 (12 to 20)	+15 (7 to 23)
18 to 30 years	68 ± 20	80 ± 23	+19 (14 to 24)	n.a.
30 to 50 years	62 ± 18	68 ± 17	+14 (5 to 24)	n.a.
≥50 years	72 ± 21	65 ± 23	–12 (–24 to 1)	n.a.
CTx, ng/L (median, IQR)	423 (323 to 533)	432 (313 to 529)	+3 (–4 to 10)	–5 (–19 to 8)
18 to 30 years	448 (384 to 590)	442 (364 to 586)	+3 (–4 to 11)	n.a.
30 to 50 years	297 (222 to 386)	313 (215 to 387)	+12 (–6 to 30)	n.a.
≥50 years	427 (305 to 547)	222 (193 to 381)	–32 (–50 to–13)	n.a.
Sclerostin, pmol/L (median, IQR)	8.7 (6.8 to 13.1)	10.3 (7.9 to 13.2)	+15 (10 to 20)	+10 (–0 to 20)
18 to 30 years	7.6 (6.5 to 9.5)	8.8 (7.4 to 11.3)	+20 (13 to 26)	n.a.
30 to 50 years	13.9 (8.8 to 16.8)	14.3 (10.8 to 18.1)	+10 (1 to 19)	n.a.
≥50 years	15.9 (14.8 to 17.8)	15.3 (13.2 to 16.3)	–10 (–19 to 0)	n.a.
*DXA*				
BMD TH g/cm^2^ (mean ± SD)	0.948 ± 0.113	0.956 ± 0.114	+0.9 (0.4 to 1.4)	+0.0 (–0.9 to 0.9)
BMD FN g/cm^2^ (mean ± SD)	0.833 ± 0.116	0.825 ± 0.116	–0.9 (–1.6 to –0.1)	–2.5 (–3.7 to–1.2)
BMD LS g/cm^2^ (mean ± SD)	1.026 ± 0.125	1.036 ± 0.129	+1.0 (0.4 to 1.7)	+2.1 (0.9 to 3.4)

HT = gender‐affirming hormonal treatment; IQR = interquartile range; ALP = alkaline phosphatase; TH = total hip; FN = femoral neck; LS = lumbar spine; n.a. = not applicable.

^a^ Adjusted for changes in BMI, alcohol and tobacco use, 25OHD, creatinine, AST, ALT, and γGT. Data only shown for the total adjusted group, as separate adjusted age groups resulted in too small groups for multivariable analyses.

### Transmen

P1NP, ALP, and sclerostin increased by 33% (95% CI, 24 to 42), 16% (95% CI, 12 to 20), and 15% (95% CI, 10 to 20), respectively, after 1 year of HT (Table 2). Adjusting these percentage changes in BTMs for changes in BMI, smoking, alcohol use, creatinine, 25OHD, AST, ALT, and γGT did not affect the results (Table 2). More detailed analyses based on the earlier specified age groups revealed an opposite effect on bone turnover in the transmen aged ≥50 years after 1 year of HT compared with younger transmen (Fig. 2). In transmen aged ≥50 years, a decrease in P1NP of –19% (95% CI, –35 to ‐4), CTx of –32% (95% CI, –50 to –13), and sclerostin of –10% (95% CI, –19 to 0) was found. Estradiol concentrations increased more in the transmen aged ≥50 years (median increase of 135 pmol/L, IQR 100 to 164) compared with transmen <50 years (median increase of 30 pmol/L, IQR –336 to 124). Different absolute concentrations of testosterone and estradiol concentrations during HT did not result in different effects in the course of BTMs during 1 year of HT (Fig. 3).

### Correlations between BTMs and BMD

Correlations between percentage change in BTMs and percentage change in BMD for transwomen and transmen are displayed in Table 3. The changes in BMD after 1 year of HT in this transgender population was described extensively in earlier research.^5^ In transwomen, an increase in sclerostin was associated with a decrease in TH BMD. No correlations between change in BTMs and FN BMD were seen. Furthermore, P1NP, ALP, and CTx showed a modest negative correlation with LS BMD after 1 year of HT. In transmen, only P1NP showed a modest negative correlation with TH and FN BMD. CTx showed a modest negative correlation with LS BMD in transmen (Table 3). Subgroup analyses were performed based on the baseline LS BMD data, which we divided into tertiles. This resulted in a mean (±SD) BMD of group 1 (0.817 ± 0.065), group 2 (0.972 ± 0.036), and group 3 (1.120 ± 0.082) in transwomen. In transmen, this resulted in a mean BMD (±SD) of group 1 (0.893 ± 0.050), group 2 (1.017 ± 0.029), and group 3 (1.167 ± 0.075). Based on these tertiles, we evaluated changes of BTMs per tertile. This resulted in a decrease of P1NP, CTx, ALP, and sclerostin in transwomen, which was similar for all tertiles. In transmen, an increase of all markers except CTx was found, which was similar for the tertiles. In CTx, an increase was found in all, but the highest BMD group (group 3).

**Table 3:** Correlation Between Percentage Change in Bone Turnover Markers and Percentage Change in BMD (mean and 95% CI), Separately for Transwomen and Transmen

Transwomen	TH BMD %	FN BMD %	LS BMD %
P1NP %	–0.10 (–0.27 to 0.09)	–0.15 (–0.32 to 0.03)	**–0.28 (–0.44** to **–0.11)**
ALP %	–0.02 (–0.19 to 0.16)	0.00 (–0.18 to 0.18)	**–0.22 (–0.38** to **–0.04)**
CTx %	–0.08 (–0.25 to 0.10)	–0.17 (–0.34 to 0.01)	**–0.27 (–0.43** to **–0.10)**
Sclerostin %	**–0.21 (–0.38** to **–0.03)**	–0.02 (–0.20 to 0.17)	0.03 (–0.15 to 0.21)

Transmen	TH BMD %	FN BMD %	LS BMD %
P1NP %	**–0.21 (–0.37** to **–0.04)**	**–0.20 (–0.36** to **–0.02)**	–0.15 (–0.32 to 0.02)
ALP %	–0.12 (–0.29 to 0.06)	–0.05 (–0.23 to 0.12)	–0.12 (–0.29 to 0.05)
CTx %	–0.09 (–0.26 to 0.09)	–0.11 (–0.28 to 0.06)	**–0.21 (–0.38** to **–0.04)**
Sclerostin %	0.08 (–0.10 to 0.25)	–0.04 (–0.21 to 0.14)	–0.08 (–0.25 to 0.10)

ALP = alkaline phosphatase.

Bold text indicate *p* ≤0.05.

## Discussion

This study evaluates changes in a variety of BTMs, sclerostin, and their correlation with changes in BMD in transgender people during the first year of HT. In transwomen, a decrease in bone turnover was seen during the first year of HT, irrespective of age. In transmen, bone turnover increased in the younger groups, and decreased in the oldest transmen. No differences were seen between the different estrogen concentrations and percentage change in BTMs. Lastly, BTMs showed some modest negative correlations with changes in the LS BMD of transwomen predominantly.

### Effects on bone turnover after one year of HT

#### Transwomen

This is the first study to evaluate sclerostin concentrations in transgender people. It is known that sclerostin concentrations are higher in men than women and sclerostin increases gradually with age in both sexes.^41^ We found that sclerostin decreased in transwomen after 1 year of HT. Previous research suggested that estrogen results in a decrease in sclerostin, which is thought to result in an increase in BMD, as sclerostin is an important inhibitor of the anabolic Wnt/β‐catenin signaling pathway in osteoblasts.^28^, ^29^ An earlier study in premenopausal estrogen‐sufficient women did not show changes in serum concentrations of sclerostin during their menstrual cycle and also did not show a relationship with estradiol concentrations.^42^ Withdrawal of estrogens, however, resulted in an increase in sclerostin in both postmenopausal women and elderly men, suggesting an inverse association between sclerostin and estrogen concentrations.^43^ A longitudinal study in Japanese women also showed a decrease in estrogen and increase in sclerostin concentrations during menopause, which resulted in increased bone resorption.^7^ The current study showed a decrease in sclerostin in transwomen after 1 year of HT. This finding provides additional evidence that estrogen treatment results in a decrease in sclerostin concentrations, which has beneficial effects on bone turnover. This finding aligns well with another study showing that treatment of postmenopausal women with the SERM raloxifene suppresses sclerostin.^44^

The finding that CTx decreased during HT is also in line with the hypothesis that the increase in estrogen concentrations reduces osteoclast activity and thereby inhibits bone resorption. Although one study found no change in CTx caused by HT in transwomen,^22^ two other studies also found a decrease in CTx concentrations within 2 years of HT and lower CTx concentrations compared with control men after 8 years of HT.^23^, ^45^ Furthermore, ALP decreased during HT. A decrease in ALP was earlier found within the first year of HT^15^ and during longer follow‐up.^12^ Earlier studies also showed a decrease in P1NP within 2 years of HT,^45^ and lower P1NP concentrations after 8 years of HT compared with control men,^23^ whereas one study showed no changes in P1NP after 3 years of HT.^22^ In addition, the lowest estradiol quartile showed opposite or even no changes in BTMs compared with the other three estradiol quartiles in transwomen, which implies that the estrogen concentrations in the lowest quartile might be too low to result in a decrease in bone turnover. Overall, the decrease in BTMs in transwomen further supports the bone‐preserving role of estrogens.

#### Transmen

Sclerostin increased in the younger transmen after 1 year of HT. The effect of androgens on sclerostin concentrations are not fully elucidated yet. An earlier study found a possible direct androgen receptor‐mediated effect on the production of sclerostin and negative correlation between sclerostin concentration and testosterone concentrations in birth‐assigned men.^46^ However, the current study did not show a decrease in sclerostin in transmen who had higher testosterone concentrations after 1 year of HT. On the other hand, another study showed that predominantly estrogen and not testosterone mediated the decrease of sclerostin.^43^ However, as both testosterone and estradiol concentrations changed in transmen, we were not able to determine the isolated effect of testosterone. Also, this result can be explained by the use of different sclerostin assays in previous literature with sometimes high variability between various sclerostin assays.^47^

With regard to bone formation, an increase in P1NP and ALP was seen after 1 year of HT. An earlier prospective study also showed an increase in P1NP with respect to no changes in control women.^24^ Furthermore, P1NP concentrations in transmen aged 37 ± 8 years were approximately 25% higher compared with control women after 10 years of HT,^24^ which is comparable to the 21% change reported in our study in this age group. Regarding ALP, earlier studies showed an increase of approximately 13% in ALP within the first year of HT in transmen aged 16 to 40 years,^15^ which is also in line with the 13% change reported in our study. A previous study in postmenopausal women showed that ALP concentrations were higher compared with premenopausal women, and that ALP was negatively correlated with the estradiol concentration of the postmenopausal group.^48^ The bone‐specific alkaline phosphatase fraction instead of total ALP is a more sensitive parameter to evaluate bone formation, as increased serum ALP can also result from liver or gallbladder disease.^38^ However, the transmen did not show signs of liver disease because all liver parameters besides ALP did not change during 1 year of HT, so it is not expected that this affected our results. As muscle mass increased in transmen, which is confirmed by an increase in creatinine after 1 year of HT, mechanical loading on bones increased, which possibly explains the increase in bone formation markers.^31^ Concerning bone resorption, the current study showed no increase in CTx in transmen after 1 year of HT. Earlier studies in transmen showed an increase in CTx after 1‐year HT compared with no changes in control women.^20^ Also, transmen had higher CTx concentrations compared with control women after 10 years of HT.^24^ CTx was measured in a fasting state, just as in the studies mentioned earlier. As CTx is cleared by the kidney,^38^ higher concentrations of CTx can be found in case of impaired kidney function, yet our study population had no impaired kidney function. Alternatively, the fasting state was based on self‐report of the participants during follow‐up. Therefore, it is possible that some participants did not apply the instructions to draw blood in a fasting state. This might have masked the increase of CTx because CTx decreases in relation to food ingestion.^38^ Lastly, when comparing age groups, the oldest transmen group showed a decrease in all BTMs and sclerostin in contrast with the younger transmen. The older group of transmen benefited the most from HT as they were assumed to be estrogen‐deficient as a result of their postmenopausal state at baseline (mean age 54 years, SD 4.1). In most studies in transmen, estradiol concentrations either remain stable or decrease slightly. However, two studies investigating the effect of testosterone in combination with an aromatase inhibitor found that estradiol concentrations remained stable in transmen using testosterone only, but decreased to a great extent in transmen using both testosterone and an aromatase inhibitor.^49^, ^50^ This indicates that the estradiol concentrations mainly result from the aromatization of testosterone into estradiol. This is also supported by our finding that estradiol concentrations increased in transmen who were postmenopausal and therefore estrogen‐deficient before the start of HT. The increase in estrogen concentration after the aromatization of testosterone resulted in decreased bone resorption, which further strengthens the beneficial role of estrogen on bone health.

### Associations between BTMs and BMD

Modest negative correlations were found between changes in BTMs and changes in BMD during 1 year of HT. This finding is in line with previous research in transwomen, where no correlations between CTx and P1NP and volumetric BMD (vBMD) of the radius or tibia where found.^22^ In transmen, only an inverse relationship with CTx and P1NP and vBMD at the radius and tibia was found.^24^ Changes in BTMs were predominantly correlated to the LS BMD of transwomen. LS consists mainly of trabecular bone, which is more metabolically active compared with the hip, which mainly consists of cortical bone.^51^, ^52^ This was not seen in the FN, although this region also contains significant trabecular bone albeit less compared with LS. This finding further emphasizes the role of estradiol in maintaining adequate bone homeostasis, which is already well‐studied in men.^53^, ^54^ Also, earlier research about estrogen supplementation therapy in postmenopausal women showed an increase in BMD and a decrease in BMD after the discontinuation of estrogen supplementation in a large female cohort.^55^ Next to this, a murine model studying ovariectomized mice showed that estrogen therapy had more beneficial effects on bone architecture compared with mice treatment with testosterone alone.^56^ Summarized, the increase in BMD after 1 year of HT in both transwomen and transmen found in this study further emphasizes the beneficial effect of estrogen on bone.

### Strengths and limitations

Data for this study were collected during patient care following a standardized treatment protocol. As a result, this prospective study consisted of a large study population compared with other studies in transgender people, thereby ensuring a study population with a broad variation in age. Other strengths of this study were^1^: the use of the same BTM assays^2^; all samples were thawed simultaneously; and 3 all analyses were performed using one lot number. Moreover, this study is the first to evaluate BTM sclerostin in transgender people. In addition, the same DXA scanner was used both at baseline and during follow‐up.

This study also has some limitations. First, no control group was included; therefore, changes in time as a cause for changes in BTMs or BMD could not be evaluated. However, as the study population consisted of different age groups, almost all participants already had reached their peak bone mass; this would have resulted in decreasing BMD through time and increased bone turnover, especially in postmenopausal women. From earlier studies, it is known that bone turnover increases with age, after the initial high levels that are reached during puberty.^57^, ^58^, ^59^ As part of standard patient care, participants were advised on the principles of a healthy lifestyle and physical activity, as well as maintaining adequate calcium and 25OHD intake. This resulted in changes in 25OHD concentrations during 1‐year HT, but adjustments for these changes did not affect our results. No full data on earlier dietary calcium intake, steroid use, fractures, weight‐bearing exercise, or family history were available. Furthermore, a 3‐month measurement of BTMs was not available. Because of the observational character of this study, this study was not designed to evaluate possible causal relationships. Nevertheless, this study further contributes to the current hypothesis that sclerostin is a mediating factor in the anabolic effect of estradiol on bone turnover and BMD. Lastly, follow‐up data regarding fractures were lacking.

To conclude, this study provides additional knowledge regarding the effect of HT on bone metabolism and BMD in transgender people and emphasizes the importance and beneficial effect of estrogen by decreasing bone turnover and increasing BMD. Summarized, this study shows that 1 year of HT does not result in deleterious effects on bone health in transgender people. Despite these results, the effects after multiple years of HT, particularly for younger transmen, are of great interest for future study. Given the still increasing incidence and the need for the treatment of transgender people, additional studies should be performed to evaluate the longer term relationships between change in bone turnover, BMD, and fracture risk during HT in transgender people.

## Disclosures

This work was supported by an unrestricted grant from Abbott Diagnostics (Chicago, IL, USA) to authors Mariska Vlot and Annemieke Heijboer. Sclerostin kits were provided by Diasorin, Saluggia, Italy. The funders had no role in the study design, data collection and analysis, or preparation of the manuscript.

## References

[1] Zamberlan N , Radetti G , Paganini C , et al. Evaluation of cortical thickness and bone density by roentgen microdensitometry in growing males and females. Eur J Pediatr. 1996;155(5):377–82. 8741034 10.1007/BF01955265

[2] Herrmann BL , Janssen OE , Hahn , et al. Effects of estrogen replacement therapy on bone and glucose metabolism in a male with congenital aromatase deficiency. Horm Metab Res. 2005;37(3):178–83. 15824973 10.1055/s-2005-861292

[3] Bilezikian JP , Morishima A , Bell J , et al. Increased bone mass as a result of estrogen therapy in a man with aromatase deficiency. N Engl J Med. 1998;339(9):599–603. 9718379 10.1056/NEJM199808273390905

[4] Carani C , Qin K , Simoni M , et al. Effect of testosterone and estradiol in a man with aromatase deficiency. N Engl J Med. 1997;337(2):91–5. 9211678 10.1056/NEJM199707103370204

[5] Wiepjes CM , Vlot MC , Klaver M , et al. Bone mineral density increases in trans persons after 1 year of hormonal treatment: a multicenter prospective observational study. J Bone Miner Res. 2017;32(6):1252–60. 28370342 10.1002/jbmr.3102

[6] Nakamura T , Imai Y , Matsumoto T , et al. Estrogen prevents bone loss via estrogen receptor alpha and induction of Fas ligand in osteoclasts. Cell. 2007;130(5):811–23. 17803905 10.1016/j.cell.2007.07.025

[7] Greendale GA , Sowers M , Han W , et al. Bone mineral density loss in relation to the final menstrual period in a multiethnic cohort: results from the Study of Women's Health Across the Nation (SWAN). J Bone Miner Res. 2012;27(1):111–8. 21976317 10.1002/jbmr.534 PMC3378821

[8] Katznelson L , Finkelstein JS , Schoenfeld DA , et al. A. Increase in bone density and lean body mass during testosterone administration in men with acquired hypogonadism. J Clin Endocrinol Metab. 1996;81(12):4358–65. 8954042 10.1210/jcem.81.12.8954042

[9] Khosla S , Melton 3rd LJ, Atkinson EJ , et al. Relationship of serum sex steroid levels and bone turnover markers with bone mineral density in men and women: a key role for bioavailable estrogen. J Clin Endocrinol Metab. 1998;83(7):2266–74. 9661593 10.1210/jcem.83.7.4924

[10] Kohrt WM , Birge SJ . Differential effects of estrogen treatment on bone mineral density of the spine, hip, wrist and total body in late postmenopausal women. Osteop Int. 1995;5(3):150–5. 10.1007/BF02106093 7655174

[11] Dittrich R , Binder H , Cupisti S , et al. Endocrine treatment of male‐to‐female transsexuals using gonadotropin‐releasing hormone agonist. Exp Clin Endocrinol Diabetes. 2005;113(10):586–92. 16320157 10.1055/s-2005-865900

[12] van Kesteren P , Lips P , Gooren LJ , et al. Long‐term follow‐up of bone mineral density and bone metabolism in transsexuals treated with cross‐sex hormones. Clin Endocrinol. 1998;48(3):347–54. 10.1046/j.1365-2265.1998.00396.x 9578826

[13] Mueller A , Haeberle L , Zollver H , et al. Effects of intramuscular testosterone undecanoate on body composition and bone mineral density in female‐to‐male transsexuals. J Sex Med. 2010;7(9):3190–8. 20584125 10.1111/j.1743-6109.2010.01912.x

[14] Lips P , van Kesteren PJ , Asscheman H , et al. The effect of androgen treatment on bone metabolism in female‐to‐male transsexuals. J Bone Miner Res. 1996;11(11):1769–73. 8915785 10.1002/jbmr.5650111121

[15] van Kesteren P , Lips P , Deville W , et al. The effect of one‐year cross‐sex hormonal treatment on bone metabolism and serum insulin‐like growth factor‐1 in transsexuals. J Clin Endocrinol Metab. 1996;81(6):2227–32. 8964856 10.1210/jcem.81.6.8964856

[16] Mueller a , Zollver H , Kronawitter D , et al. Body composition and bone mineral density in male‐to‐female transsexuals during cross‐sex hormone therapy using gonadotrophin‐releasing hormone agonist. Exp Clin Endocrinol Diabetes. 2011;119(2):95–100. 20625973 10.1055/s-0030-1255074

[17] Singh‐Ospina N , Maraka S , Rodriguez‐Gutierrez R , et al. Effect of sex steroids on the bone health of transgender individuals: a systematic review and meta‐analysis. J Clin Endocrinol Metab. 2017;102(11):3904–13. 28945851 10.1210/jc.2017-01642

[18] Vlot MC , Klink DT , den Heijer M , et al. Effect of pubertal suppression and cross‐sex hormone therapy on bone turnover markers and bone mineral apparent density (BMAD) in transgender adolescents. Bone. 2017;95:11–9. 27845262 10.1016/j.bone.2016.11.008

[19] Wiepjes CM , de Jongh RT , de Blok CJM , et al. Bone safety during the first ten years of gender‐affirming hormonal treatment in transwomen and transmen. J Bone Miner Res. 2019;34(3):447–54. 30537188 10.1002/jbmr.3612 PMC7816092

[20] Van Caenegem E , Wierckx K , Taes Y , et al. Body composition, bone turnover, and bone mass in trans men during testosterone treatment: 1‐year follow‐up data from a prospective case‐controlled study (ENIGI). Europ. J Endocrinol. 2015;172(2):163–71. 10.1530/EJE-14-0586 25550352

[21] Sosa M , Jodar E , Arbelo E , et al. Bone mass, bone turnover, vitamin D, and estrogen receptor gene polymorphisms in male to female transsexuals: effects of estrogenic treatment on bone metabolism of the male. J Clin Densitom. 2003;6(3):297–304. 14515001 10.1385/jcd:6:3:297

[22] T’Sjoen G , Weyers S , Taes Y , et al. Prevalence of low bone mass in relation to estrogen treatment and body composition in male‐to‐female transsexual persons. J Clin Densitom. 2009;12(3):306–13. 19121966 10.1016/j.jocd.2008.11.002

[23] Lapauw B , Taes Y , Simoens S , et al. Body composition, volumetric and areal bone parameters in male‐to‐female transsexual persons. Bone. 2008;43(6):1016–21. 18835591 10.1016/j.bone.2008.09.001

[24] Van Caenegem E , Wierckx K , Taes Y , et al. Bone mass, bone geometry, and body composition in female‐to‐male transsexual persons after long‐term cross‐sex hormonal therapy. J Clin Endocrinol Metab. 2012;97(7):2503–11. 22564669 10.1210/jc.2012-1187

[25] Zucker KJ . Epidemiology of gender dysphoria and transgender identity. Sex Health. 2017;14(5):404–11. 28838353 10.1071/SH17067

[26] Wiepjes CM , Nota NM , de Blok CJM , et al. The Amsterdam Cohort of Gender Dysphoria Study (1972–2015): Trends in prevalence, treatment, and regrets. J Sex Med. 2018;15(4):582–90. 29463477 10.1016/j.jsxm.2018.01.016

[27] Matsui S , Yasui T , Kasai K , et al. Increase in circulating sclerostin at the early stage of menopausal transition in Japanese women. Maturita. 2016;83:72–7. 10.1016/j.maturitas.2015.10.001 26508082

[28] Krishnan V , Byrant HU , MacDougald OA . Regulation of bone mass by Wnt signaling. J Clin Invest. 116(5):1202–9. 10.1172/JCI28551 PMC1451219 16670761

[29] Jia HB , Ma JX , Ma XL , et al. Estrogen alone or in combination with parathyroid hormone can decrease vertebral MEF2 and sclerostin expression and increase vertebral bone mass in ovariectomized rats. Osteoporos Int. 2014;25(12):2743–54. 25074352 10.1007/s00198-014-2818-y

[30] Compton JT , Lee FY . A review of osteocyte function and the emerging importance of sclerostin. J Bone Joint Surg Am. 2014;96(19):1659–68. 25274791 10.2106/JBJS.M.01096 PMC4179450

[31] Delgado‐Calle J , Tu X , Pacheco‐Costa R , et al. Control of bone anabolism in response to mechanical loading and PTH by distinct mechanisms downstream of the PTH receptor. J Bone Miner Res. 2017;32(3):522–35. 27704638 10.1002/jbmr.3011 PMC8502039

[32] Suen PK , Qin L . Sclerostin, an emerging therapeutic target for treating osteoporosis and osteoporotic fracture: a general review. J Orthop Transl. 2016;4:1–13. 10.1016/j.jot.2015.08.004 PMC5987014 30035061

[33] Dekker MJHJ , Wierckx K , Van Caenegem E , et al. European network for the investigation of gender incongruence: Endocrine part. J Sex Med. 2016;13(6):994–9. 27162190 10.1016/j.jsxm.2016.03.371

[34] Kreukels BPC , Haraldsen IR , De Cuypere G , et al. European network for the investigation of gender incongruence: the ENIGI initiative. Eur Psychiatry. 2012;27(6):445–50. 20620022 10.1016/j.eurpsy.2010.04.009

[35] American Psychiatric Association . Diagnostic and statistical manual of mental disor‐ ders. 4th ed Arlington, VA: American Psychiatric Association; 2000: pp 576–81.

[36] American Psychiatric Association . Diagnostic and statistical manual of mental disorders. 5th ed (DSM‐5). Arlington, VA: American Psychiatric Publishing; 2013.

[37] The World Professional Association for Transgender Health. Standards of care for the health of transsexual, transgender, and gender‐ nonconforming people. Version 7. Elgin, IL: World Professional Association for Transgender Health; 2012.

[38] Vlot MC , den Heijer M , de Jongh RT , et al. Clinical utility of bone markers in various diseases. Bone. 2018;114:215–25. 29920402 10.1016/j.bone.2018.06.011

[39] Heijboer AC , Blankenstein MA , Kema IP , et al. Accuracy of 6 routine 25‐hydroxyvitamin D assays: influence of vitamin D binding protein concentration. Clin Chem. 2012;58(3):543–8. 22247500 10.1373/clinchem.2011.176545

[40] Dirks NF , Vesper HW , van Herwaarden AE , et al. Various calibration procedures result in optimal standardization of routinely used 25(OH)D ID‐LC‐MS/MS methods. Clin Chim Acta. 2016;462:49–54. 27570062 10.1016/j.cca.2016.08.016 PMC5703036

[41] Clarke BL , Drake MT . Clinical utility of serum sclerostin measurements. Bonekey Rep. 2013;2(JUNE):361. 24578825 10.1038/bonekey.2013.95 PMC3936109

[42] Liakou CG , Mastorakos G , Makris K , et al. Changes of serum sclerostin and Dickkopf‐1 levels during the menstrual cycle. A pilot study. Endocrine. 2016;54(2):543–51. 27601021 10.1007/s12020-016-1056-9

[43] Modder UIl , Clowes JA , Hoey K , et al. Regulation of circulating sclerostin levels by sex steroids in women and in men. J Bone Miner Res. 2011;26(1):27–34. 20499362 10.1002/jbmr.128 PMC3132405

[44] Chung YE , Lee SH , Lee SY , et al. Long‐term treatment with raloxifene, but not bisphosphonates, reduces circulating sclerostin levels in postmenopausal women. Osteoporos Int. 2012;23(4):1235–43. 21660558 10.1007/s00198-011-1675-1

[45] Van Caenegem E , Wierckx K , Taes Y , et al. Preservation of bone mass in trans women during cross‐sex hormonal therapy: a prospective observational study. Osteoporos Int. 2014;26(1):35–47. 25377496 10.1007/s00198-014-2805-3

[46] Di Nisio A , De Toni L , Speltra E , et al. Regulation of sclerostin production in human male osteocytes by androgens. Endocrinology. 2015;156(12):4534–44. 26393301 10.1210/en.2015-1244

[47] Piec I , Washbourne C , Tang J , et al. How accurate is your sclerostin measurement? Comparison between three commercially available sclerostin ELISA kits. Calcif Tissue Int. 2016;98(6):546–55. 26749312 10.1007/s00223-015-0105-3 PMC4860200

[48] Pardhe BD , Pathak S , Bhetwal A , et al. Effect of age and estrogen on biochemical markers of bone turnover in postmenopausal women: a population‐based study from Nepal. Int J Womens Health. 2017;9:781–8. 29123427 10.2147/IJWH.S145191 PMC5661842

[49] Bunck MC , Toorians AW , Lips P , et al. The effect of the aromatase inhibitor anastrozole on bone metabolism and cardiovascular risk indices in ovariectomized, androgen‐treated female‐to‐male transsexuals. Eur J Endocrinol. 2006;154:569–75. 16556720 10.1530/eje.1.02126

[50] Meriggiola MC , Armilotta F , Costantino A , et al. Effects of testosterone undecanoate administered alone or in combination with letrozole or dutasteride in female to male transsexuals. J Sex Med. 2008;5(10):2442–53. 18624972 10.1111/j.1743-6109.2008.00909.x

[51] Lehtonen‐Veromaa M , Mottonen T , Irjala K , et al. A 1‐year prospective study on the relationship between physical activity, markers of bone metabolism, and bone acquisition in peripubertal girls. J Clin Endocrinol Metab. 2000;85(10):3726–32. 11061531 10.1210/jcem.85.10.6889

[52] Tracz MJ , Sideras K , Bolona ER , et al. Testosterone use in men and its effects on bone health. A systematic review and meta‐analysis of randomized placebo‐controlled trials. J Clin Endocrinol Metab. 2006;91(6):2011–6. 16720668 10.1210/jc.2006-0036

[53] Cauley JA , Ewing SK , Taylor BC , et al. Sex steroid hormones in older men: longitudinal associations with 4.5‐year change in hip bone mineral density—The Osteoporotic Fractures in Men Study. J Clin Endocrinol Metab. 2010;95(9):4314–23. 20554716 10.1210/jc.2009-2635 PMC2936055

[54] Finkelstein JS , Lee H , Leder BZ , et al. Gonadal steroid—dependent effects on bone turnover and bone mineral density in men. J Clin Invest. 2016;126(3):1114–25. 26901812 10.1172/JCI84137 PMC4767351

[55] Skouby SO , Al‐Azzawi F , Barlow D , et al. Climacteric medicine: European Menopause and Andropause Society (EMAS) 2004/2005 position statements on peri‐ and postmenopausal hormone replacement therapy. Maturitas. 2005;51(1):8–14. 15883103 10.1016/j.maturitas.2005.02.019

[56] Goetz LG , Mamillapalli R , Devlin MJ , et al. Cross‐sex testosterone therapy in ovariectomized mice: addition of low‐dose estrogen preserves bone architecture. Am J Physiol Endocrinol Metab. 2017;313(5):E540–51. 28765273 10.1152/ajpendo.00161.2017 PMC5792142

[57] Sone T , Miyake M , Takeda N , et al. Urinary excretion of type I collagen crosslinked N‐telopeptides in healthy Japanese adults: age‐ and sex‐related changes and reference limits. Bone. 1995;17(4):335–9. 8573404 10.1016/s8756-3282(95)00243-x

[58] Blumsohn A , Hannon RA , Wrate R , et al. Biochemical markers of bone turnover in girls during puberty. Clin Endocrinol (Oxf). 1994;40(5):663–70. 7516828 10.1111/j.1365-2265.1994.tb03019.x

[59] Walsh JS , Henry YM , Fatayerji D , et al. Hormonal determinants of bone turnover before and after attainment of peak bone mass. Clin Endocrinol (Oxf). 2010;72(3):320–7. 19508592 10.1111/j.1365-2265.2009.03606.x

